# Multiband Signal Receiver by Using an Optical Bandpass Filter Integrated with a Photodetector on a Chip

**DOI:** 10.3390/mi14020331

**Published:** 2023-01-27

**Authors:** Xiuyou Han, Meng Chao, Xinxin Su, Weiheng Wang, Shuanglin Fu, Zhenlin Wu, Mingshan Zhao

**Affiliations:** School of Optoelectronic Engineering and Instrumentation Science, Dalian University of Technology, Dalian 116024, China

**Keywords:** photonic integration, microwave photonics, multiband RF signal receiver, silicon photonic chip

## Abstract

Photonic integration brings the promise of significant cost, power and space savings and propels the real applications of microwave photonic technology. In this paper, a multiband radio frequency (RF) signal simultaneous receiver using an optical bandpass filter (OBPF) integrated with a photodetector (PD) on a chip is proposed, which was experimentally demonstrated. The OBPF was composed of ring-assisted Mach–Zehnder interferometer with a periodical bandpass response featuring a box-like spectral shape. The OBPF was connected to a PD and then integrated onto a single silicon photonic chip. Phase-modulated multiband RF signals transmitted from different locations were inputted into the OBPF, by which one RF sideband was filtered out and the phase modulation to intensity modulation conversion was realized. The single sideband with carrier signals were then simultaneously detected by the PD. A proof-of-concept experiment with the silicon photonic integrated chip was implemented to simultaneously receive four channels of 8 GHz, 12 GHz, 14 GHz and 18 GHz in the X- and Ku-bands. The performance of the integrated microwave photonic multiband receiver—including the receiving sensitivity, the spurious free dynamic range, the gain and the noise figure across the whole operation frequency band—was characterized in detail.

## 1. Introduction

Microwave photonics technology brings together the worlds of radio frequency (RF) engineering and optoelectronics [1,2], exhibiting a promising potential in applications such as broadband wireless access networks [3], satellite communications [4], radar [5] and electronic warfare systems [6]. Photonic integration enables a dramatic reduction in the footprint of microwave photonics systems with a high complexity and enhances functionalities and performance, propelling it into real applications [7,8]. Multiband RF signal transmitting, processing and receiving with microwave photonics technology has attracted more and more attention in new generation wireless communication systems [9,10], photonic-aided payloads for high-throughput satellites [11,12] and “software-defined” radar with multifunctions of sensing, tracking and imaging [13,14].

With the rapid development of photonic integration technology [15,16], chip-based photonic multiband RF signal processing has shown an effective reduction in the size, weight and power consumption (SWaP) as well as the capability of enhancing the system performance. In 2017, an integrated photonic-assisted full-band tunable RF transceiver based on a silicon-on-insulator (SOI) platform was proposed by Chen et al. [17]. The measured results demonstrated the multiple signal processing function of up/down-conversions, phase shifting and filtering as well as the frequency multiplier of the local oscillator. In 2020, a silicon chip-based microwave photonic radar for high-resolution imaging was first demonstrated experimentally by Pan et al. [18], with the photonic frequency-doubled linear frequency modulation signal generator and the photonic de-chirp receiver covering the full Ku-band (12–18 GHz). In 2022, the first outdoor field trial of a coherent dual-band photonics-based radar was implemented through packaged silicon photonic integrated circuits by Bogoni et al. [19], demonstrating the full functionality of such a multiband radar system. It operated in both the S- and X-bands, with an operative detection capability suitable for real maritime traffic.

In this paper, a multiband RF signal simultaneous receiver using an optical bandpass filter (OBPF) integrated with a photodetector (PD) on a chip is proposed and experimentally demonstrated. Phase-modulated multiband RF signals transmitted from different locations were inputted into the OBPF with a periodical box-like bandpass filtering response. One sideband of the RF signals, optically carried by different wavelengths, was filtered out and single sideband with carrier (SSB + C) signals were correspondingly selected by the passband of the OBPF. The SSB + C signals were then simultaneously detected by one PD. The receiving scheme with the phase modulation of the RF signal and optical sideband filtering avoided the complex bias control needed for conventional intensity modulation and had immunity to the dispersion-induced power penalty for high-frequency RF signals over a long-distance transmission. A proof-of-concept experiment with the silicon photonic integrated chip was implemented to simultaneously receive four channels of 8 GHz, 12 GHz, 14 GHz and 18 GHz, covering the X- and Ku-bands. The performance of the integrated microwave photonic multiband receiver—including the receiving sensitivity, the spurious free dynamic range (SFDR), the gain and the noise figure (NF) across the whole operation frequency band—was measured and analyzed in detail.

## 2. Architecture and Operation Principle of Multiband RF Signal Receiver

**Figure 1:**
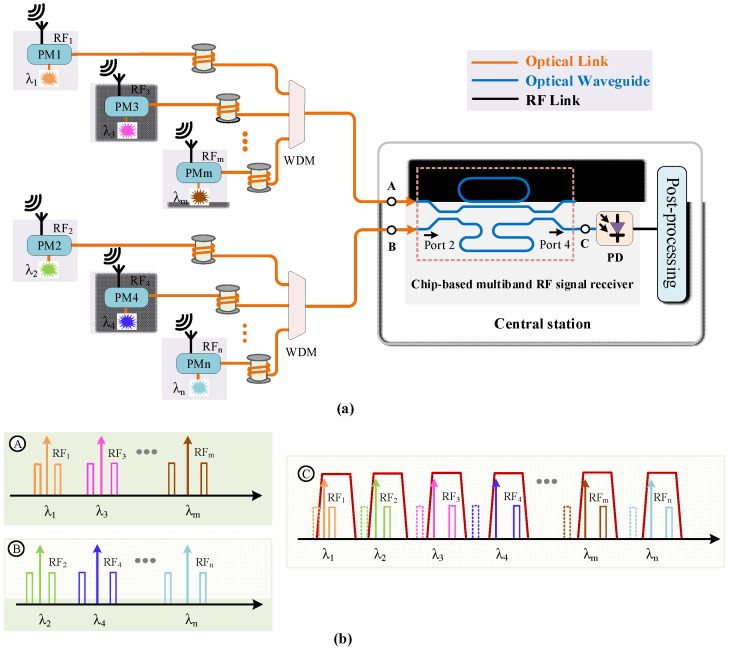
(**a**) Schematic of multiband RF signal receiving system; (**b**) spectral illustration of phase modulation signals at point A and B and sideband filtering via OBPF at point C. PM: phase modulator; WDM: wavelength division multiplexer; OBPF: optical bandpass filter; PD: photodetector.

The proposed multiband RF signal receiver is shown in Figure 1. The different RF band signals received by the antennas located on the designated sites were phase-modulated on the optical carriers, with the corresponding wavelength aligned to the passband of the OBPF. The phase-modulated RF signals from the different sites were transmitted via optical fibers and were combined via a wavelength division multiplexer (WDM) with the central station where the OBPF and the PD were integrated on one chip as the channel selector and detector, respectively. The OBPF was composed of a Mach–Zehnder interferometer (MZI) assisted by a ring resonator exhibiting a box-like passband and a periodical filtering response [20]. One sideband of the phase-modulated RF signal was filtered out by the OBPF and the conversion of phase modulation to intensity modulation was realized. The SSB + C signal was then inputted into the PD. The RF signal was recovered by frequency beating between the sideband and the optical carrier on the PD. The following gives the deduction of the phase modulation, sideband filtering and detection.

The RF signal received by the antenna was modulated to the optical carrier from the laser source via the phase modulator. The output could be expressed as [21]

(1) $$E_{out-i}=E_{O-i}e^{j2\pi f_{O-i}t}e^{j\pi \frac{V_{RF-i}\cos(2\pi f_{RF-i}t)}{V_{\pi i}}}$$

where *E*_out-*i*_ and *f*_O-*i*_ are the amplitude and frequency, respectively, of the optical carrier from the laser source at site *i*; *V*_RF-*i*_ and *f*_RF-*i*_ are the voltage value and frequency of the RF signal, respectively; and *V*_π-*I*_ is the half-wave voltage of the *i^th^* phase modulator. By using Jacobi–Anger expansions, Equation (1) could be expanded:

(2) $$E_{out-i}=E_{O-i}\left\{J_{1}\left(m_{1-i}\right)e^{j\left[2\pi \left(f_{O-i}-f_{RF-i}\right)t-\pi /2\right]}+J_{0}\left(m_{1-i}\right)e^{j2\pi f_{O-i}t}+J_{1}\left(m_{1-i}\right)e^{j\left[2\pi \left(f_{O-i}+f_{RF-i}\right)t+\pi /2\right]}\right\}$$

where *J_0_* and *J*_1_ are the 0- and 1st-order Bessel function of the first kind. When deriving Equation (2), only the 0- and ± 1st-order components were considered; the higher-order ones were ignored, which is suitable for small signal modulations. The schematic spectral output from the phase modulators are shown as the insets at point A and point B in Figure 1. From Equation (2) it could be seen that there was a π phase difference between the + 1st-order sideband and the −1st-order sideband. If the phase-modulated optical signal was inputted into the PD directly for detection, the beating signal between the + 1st-order sideband and the optical carrier cancelled the beating one between the −1st-order sideband and the optical carrier. There was no signal to be obtained [21]. The phase-modulated signal transmitted to the OBPF and one sideband was filtered out, as shown in the inset at point C of Figure 1. The relationship of the π phase difference between the ± 1st-order sidebands was broken and the conversion from phase modulation to intensity modulation was realized. The SSB + C signal was obtained and could be expressed as

(3) $${E^{\prime }}_{out-i}=E_{O-i}\left\{J_{0}\left(m_{1-i}\right)e^{j2\pi f_{O-i}t}+J_{1}\left(m_{1-i}\right)e^{j\left[2\pi \left(f_{O-i}+f_{RF-i}\right)t+\pi /2\right]}\right\}$$

The RF signal was recovered by the O/E conversion of the SSB + C signal on the PD and could be expressed as

(4) $$I_{RF-i}\propto ac\left(\rho \cdot {E^{\prime }}_{out-i}\cdot {E^{\prime }}_{out-i}^{*}\right)=2\rho E_{O-i}^{2}J_{0}\left(m_{1-i}\right)J_{1}\left(m_{1-i}\right)\cos\left(2\pi f_{RF-i}t+\pi /2\right)$$

where *ρ* is the responsivity of the PD. The above deduction was conducted for the *i^th^* antenna; it was also applicable to all other antennas in the receiving system. One sideband of each phase-modulated signal was simultaneously filtered out and the SSB + C signals were outputted from the OBPF.

For the OBPF, according to the matrix transmission method, the filtering response of the ring-assisted MZI structure could be expressed as [22]

(5) $$\left[\begin{array}{c} E_{3}\\ E_{4} \end{array}\right]=\left[\begin{array}{cc} \sqrt{1-\kappa_{2}} & -j\sqrt{\kappa_{2}}\\ -j\sqrt{\kappa_{2}} & \sqrt{1-\kappa_{2}} \end{array}\right]\left[\begin{array}{cc} H_{R} & 0\\ 0 & e^{-j\Delta \theta } \end{array}\right]\left[\begin{array}{cc} \sqrt{1-\kappa_{1}} & -j\sqrt{\kappa_{1}}\\ -j\sqrt{\kappa_{1}} & \sqrt{1-\kappa_{1}} \end{array}\right]\left[\begin{array}{c} E_{1}\\ E_{2} \end{array}\right]$$

where *κ*_1_ and *κ*_2_ are the intensity cross-coupling coefficients of the couplers in the MZI; Δ*θ* is the phase difference between the two arms of the MZI, which was the combination of the waveguide length difference-induced phase and the additional phase by the phase shifter on the lower arm; and H_R_ is the transmission function of the ring resonator [23] on the upper arm of the MZI, which provided the phase compensation to accelerate the phase change in the MZI. By optimizing the phase shift at different points in the MZI, a box-like bandpass response could be realized. The output responses of Port 3 and Port 4 of the ring-assisted MZI structure were complementary to each other when Port 1 or Port 2 was used as the input port. Namely, the passband of Port 3 corresponded with the stopband of Port 4 and vice versa. Therefore, Port 1 and Port 2 could be utilized as the input ports of the OBPF, which multiplexed the phase-modulated signals to Port 4 with the output of the SSB + C signals, as illustrated in the inset of the schematic spectra at point C. The output of the SSB + C signals were inputted into the PD, upon which the multiband RF signals could be simultaneously detected. There was no special requirement for the optically carried RF bands to proceed to the specific input port of the ring-assisted MZI OBPF. The optical carrier frequency spacing between the adjacent channels could be one or integral multiple half-free spectrum ranges (FSRs) of the OBPF, as long as it was larger than the frequency response range of the PD, which eliminated the crosstalk of the multiple channels during detection. The ring-assisted MZI-based OBPF combined the functions of sideband filtering and channel multiplexing. The multiband RF signals could then be detected by one PD, which greatly enhanced the compactness of the receiver.

## 3. Photonic Integrated Chip Fabrication and Experimental Results

### 3.1. Silicon Photonic Integrated Chip

**Figure 2:**
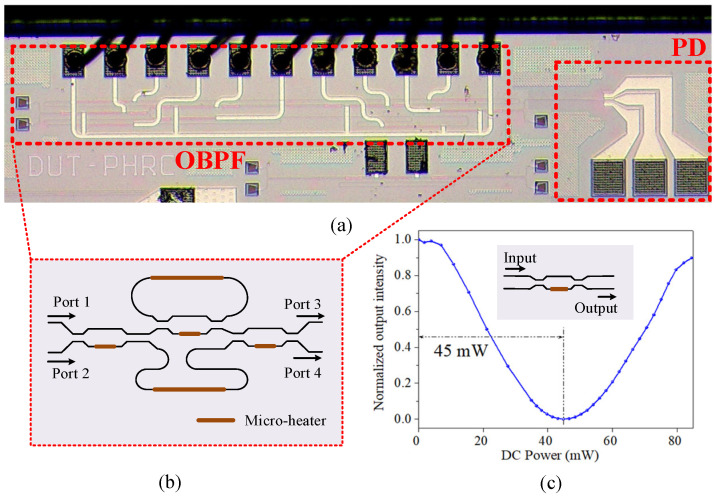
(**a**) The optical microscope image of the photonic integrated chip, including the OBPF and PD; (**b**) schematic of the OBPF; (**c**) MZI-based tunable coupler test.

The multiband RF signal receiver, including the OBPF and the PD, was fabricated on a standard 220 nm silicon-on-insulator platform. In order to meet the single-mode transmission of the 1550 nm wavelength, the width of the passive strip waveguide was designed to be 450 nm. Figure 2a shows the optical microscope images of the photonic integrated chip and Figure 2b illustrates the schematic of the designed OBPF. The coupling coefficients of the couplers in the OBPF based on the sub-MZI structure were tunable [24]. The sub-MZI structure, with a TiN micro-heater on one arm as the phase shifter, was utilized as the tunable coupler for the coupling coefficient tuning in the OBPF. The performance of the tunable coupler on the same chip was tested. Figure 2c shows the relative output intensity of the tunable coupler with the DC power applied to the micro-heater. It can be seen from Figure 2c that the coupling coefficient could be tuned from maximum to minimum with a power consumption of 45 mW.

**Figure 3:**
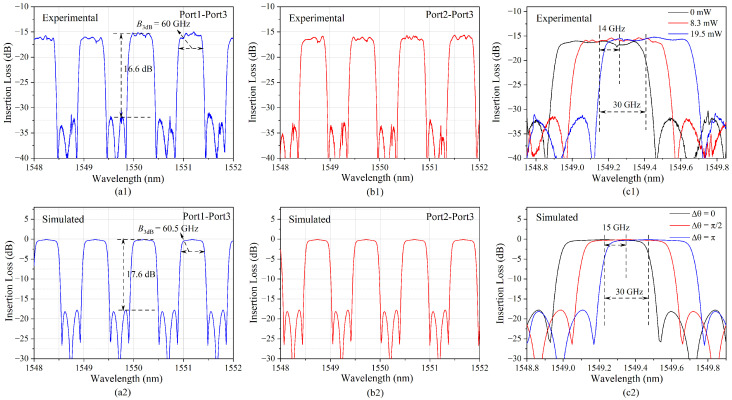
(**a1**,**a2**) Filtering response from Port 1 to Port 3 and (**b1**,**b2**) from Port 2 to Port 3; (**c1**,**c2**) filtering response from Port 1 to Port 3 by changing the applied DC voltage on the micro-heater on the ring and the MZI arm. The upper figures of (**a1**,**b1**,**c1**) are the experimental results; the lower figures of (**a2**,**b2**,**c2**) are the simulated results.

The filtering response of the OBPF was the characterized with an optical vector analyzer (OVA, LUNA 5000, Roanoke, Virginia, USA). The phase shift at different points in the ring-assisted MZI structure was optimized by changing the applied DC voltage on the micro-heater and the measured optical spectra from Port 1 to Port 3—namely, the bar state—and from Port 2 to Port 3—namely, the cross state; these are plotted in Figure 3a1,b1, respectively. The simulated filtering response was also plotted (Figure 3a2,b2). It could be seen that the measured curves matched well with the designed ones. The box-like bandpass response was realized with a 3 dB bandwidth of about 0.48 nm (~60 GHz), an out-of-band suppression of about 16.6 dB and a FSR of 123 GHz. By comparing Figure 3a1,b1, the complementary filtering response between the bar state and cross state was demonstrated. The maximum signal frequency that could be received was theoretically determined by the 3 dB bandwidth of the OBPF; namely, 60 GHz for the fabricated OBPF chip. The periodical bandpass filtering property of the OBPF provided the capacity of avoiding crosstalk for multiple channel receiving, as long as the optical carrier frequency spacing between the adjacent channels was higher than the frequency response range of the PD. The central wavelength of the passband could be adjusted by changing the DC power on the micro-heaters on the ring and the MZI arm. Figure 3c1 shows the measured filtering response of Port 3 when the light wave was inputted via Port 1 with DC power of 0 mW, 8.3 mW and 19.5 mW. It could be seen that the central wavelength adjusted to about 0.24 nm (30 GHz) with the DC power of 19.5 mW; the box-like spectral shape was well-maintained. It verified the feasibility of the OBPF to be used as the sideband filter when the operation RF band changed. According to the operation principle of the ring-assisted MZI filter [20], for the central wavelength shift of a quarter of the FSR, a π phase shift was applied to the micro-ring, which was basically consistent with the simulation results shown in Figure 3c2.

**Figure 4:**
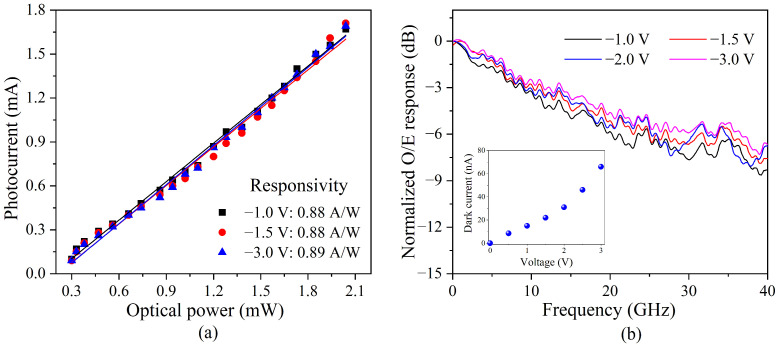
(**a**) Measured responsivity and (**b**) normalized O/E response of a PD on a chip.

The performance of the PD on a chip was measured. In order to determine the responsivity of the PD, the fiber-to-waveguide coupling loss via grating and the transmission loss via the straight waveguide were tested for the neighboring waveguides on the chip. The fiber-to-waveguide coupling loss was 6.17 dB and the transmission loss of the waveguide with a length of 10 μm was about 0.002 dB, according to the measurement of the long waveguide on the same chip. A reverse voltage bias was applied to the device through a bias tee. The current source (Keithley 2602A, Beaverton, Oregon, USA) recorded the photocurrents at different inputted optical powers at a wavelength of 1550 nm; the results are shown in Figure 4a. It could be seen that the responsivity at different reverse voltages of −1.0 V, −1.5 V and −3.0 V were 0.88 A/W, 0.88 A/W and 0.89A/W, respectively.

The RF detection response of the PD was tested by using a vector network analyzer (VNA)-based measurement system [25]. A RF signal from Port 1 of the VNA (Keysight, N5247B, Santa Rosa, CA, USA) was modulated on the optical carrier via an external intensity modulator with a bandwidth of 40 GHz. The RF-modulated optical signal was coupled to the device and the electrical output was measured through a high-speed RF probe to Port 2 of the VNA. The frequency response of the PD at different reverse bias voltages is shown in Figure 4b. Among them, the frequency response of the RF cable, bias tee and intensity modulator were removed. It could be seen that the bandwidth of the PD gradually increased as the reverse bias voltage increased. The inset in Figure 4b provides the measured dark current of the PD at different reverse bias voltages, showing the increased dark current with the enhancement of the reverse bias voltage. The thermal noise due to the dark current deteriorated the noise figure merit of the RF signal receiving system. In order to obtain a relatively large bandwidth and a low dark current, the reverse bias voltage for the PD was set at −1.5 V in subsequent test with a 3 dB bandwidth of 11 GHz, a 6 dB bandwidth of 25 GHz and a dark current of 22 nA.

### 3.2. Multiband RF Signal Receiver

**Figure 5:**
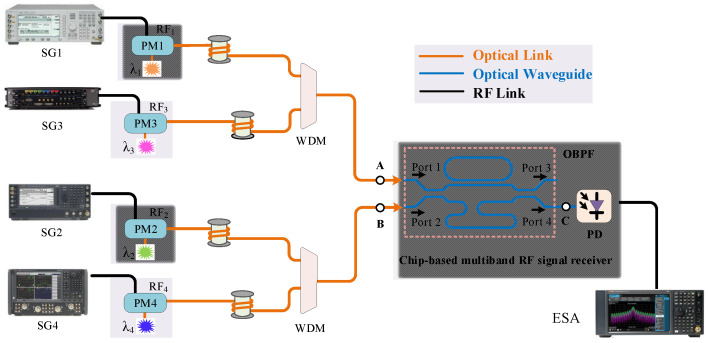
Experimental setup of the proposed multiband RF signal simultaneous receiver. SG: signal generator; ESA: electrical spectrum analyzer.

**Figure 6:**
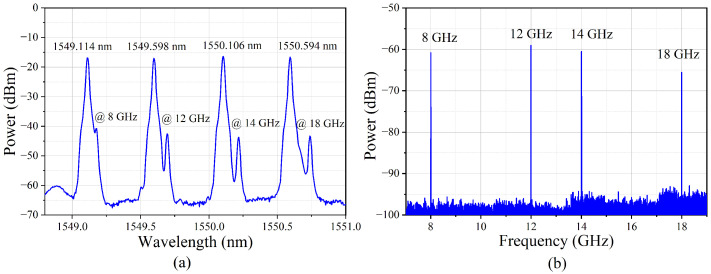
(**a**) The optical spectra of the four channels after OBPF. (**b**) The electrical spectra of the four channels of single-frequency-modulated signal after PD.

A proof-of-concept experiment of the fabricated on-chip multiband RF signal receiver was carried out. Four channels of 8 GHz, 12 GHz, 14 GHz and 18 GHz covering the X- and Ku-bands were tested by the experimental setup shown in Figure 5. The wavelengths of the four laser sources (Emcore DFB-1782A, Albuquerque, New Mexico, USA, NKT Photonics Koheras ADJUSTIK POWER E15 K81-152-04, NKT Photonics Koheras ADJUSTIK POWER E15 K82-152-13, Birkerød, Denmark, and Tengguang DFB-1550-F-S-1-PM, Mianyang, Sichuan, China) were set as λ_1_ = 1549.114 nm, λ_2_ = 1549.598 nm, λ_3_ = 1550.106 nm and λ_4_ = 1550.594 nm, respectively. The RF signals of different frequencies (8 GHz, 12 GHz, 14 GHz and 18 GHz) from four signal generators (Agilent E8267D, Keysight E8257D, Keysight M8196A and Keysight N5247B, Santa Rosa, CA, USA) were then modulated on the optical carriers via four phase modulators (EOSPACE PM-DV5-40, Redmond, WA, USA), respectively. The phase-modulated RF signals carried by λ_1_ and λ_3_ were combined and inputted into Port 1 and the phase-modulated RF signals carried by λ_2_ and λ_4_ were combined and inputted into Port 2. Figure 6a shows the optical spectra output from the OBPF measured by the optical spectrum analyzer (OSA, YOKOGAWA AQ6370D, Tokyo, Japan). It could be seen that the sideband filtering of the four channels was completed and the SSB + C signals were obtained. Figure 6b shows the electrical spectra output from the PD measured by the electrical spectrum analyzer (ESA, Keysight N9030B, Santa Rosa, CA, USA), which demonstrated that the simultaneous receiving of multiband RF signals was successfully realized.

**Figure 7:**
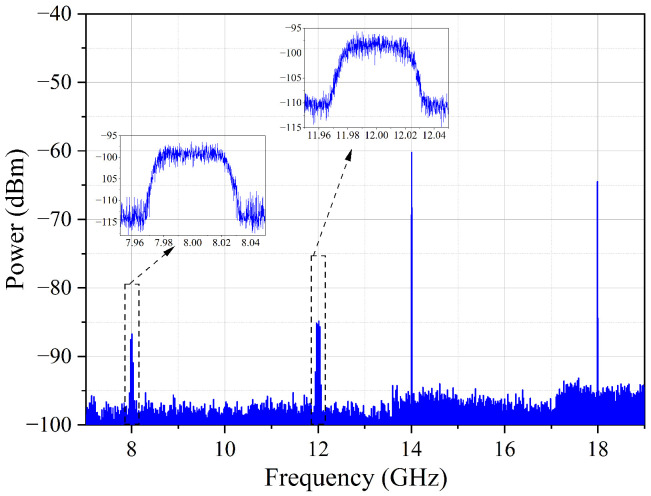
The electrical spectra of the four channels with two channels modulated by 16 QAM signal of 50 Msps. RBW: 10 kHz; VBW: 10 kHz; span: 12 GHz. The insets are the zoom-in spectra of Channel-1 and Channel-2, which were averaged 10 times during the measurement. RBW: 10 kHz; VBW: 10 kHz; span: 100 MHz.

**Figure 8:**
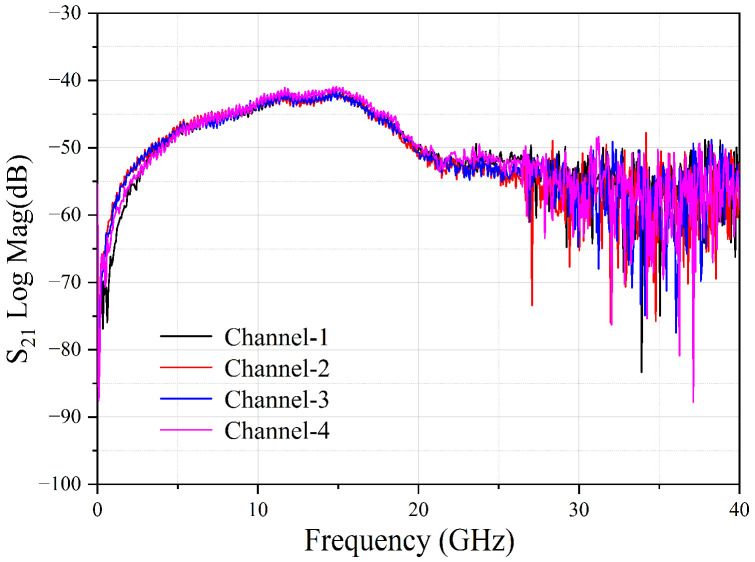
The RF receiving response of the four channels.

The receiving capacity of the modulated RF signals with a certain bandwidth was also verified. Two channels, limited by the current equipment to generate modulation signals in a laboratory (Channel-1 of 8 GHz and Channel-2 of 12 GHz), were selected to carry a 16 QAM signal with a bandwidth of 50 Msps. The measured spectrum is shown in Figure 7, where the insets are the zoom-in spectra of the modulated signals at 8 GHz and 12 GHz, respectively. The difference between the signal power and the noise floor of the inset spectra was due to the different settings of the electrical spectrum analyzer during the test. From Figure 7, it could be seen that there were only the received RF signals and no spurious components, reflecting no crosstalk between the channels. Figure 8 shows the receiving RF response of the four channels. Considering the 6 dB bandwidth response, the photonic chip-based system could receive RF signals from 5 GHz to 19 GHz covering the C-, X- and Ku-bands.

### 3.3. MWP Receiving System Performance

**Figure 9:**
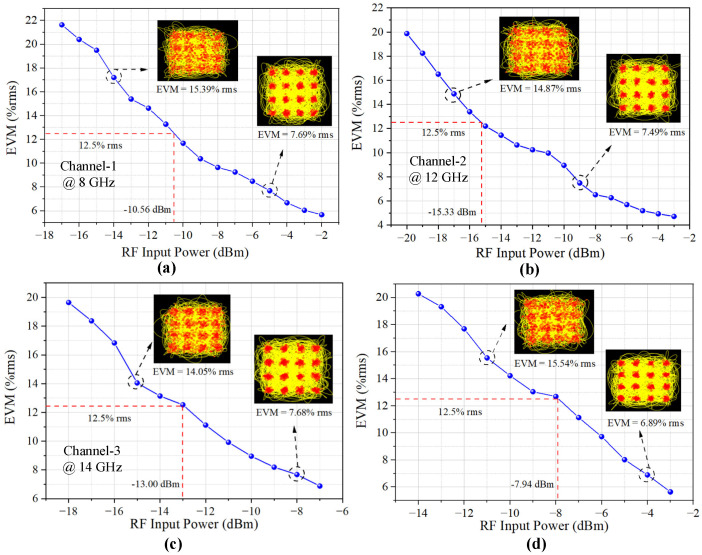
Receiving sensitivity of the photonic chip-based MWP system for four channels at (**a**) 8 GHz, (**b**) 12 GHz, (**c**) 14 GHz and (**d**) 18 GHz.

The performance of the multichannel MWP receiving system, including the sensitivity, SFDR, gain and NF, were tested and analyzed in detail. First, the receiving sensitivity of the four channels was measured. During the test, the power of the modulated signal of the 16 QAM with a bandwidth of 50 Msps at different frequencies was gradually changed and the error vector magnitude (EVM) value and constellation diagram of the received signal were measured; the results are shown in Figure 9. According to the 3-GPP standard [26], an EVM value of 12.5% is the threshold of the 16 QAM signal. The receiving sensitivity of Channel-1 (@ 8 GHz), Channel-2 (@ 12 GHz), Channel-3 (@ 14 GHz) and Channel-4 (@ 18 GHz) were −10.56 dBm, −15.33 dBm, −13.00 dBm and −7.94 dBm, respectively. When the power of the received signal was larger than the threshold, the constellation diagram exhibited a gathered state and the signal could successfully be demodulated.

**Figure 10:**
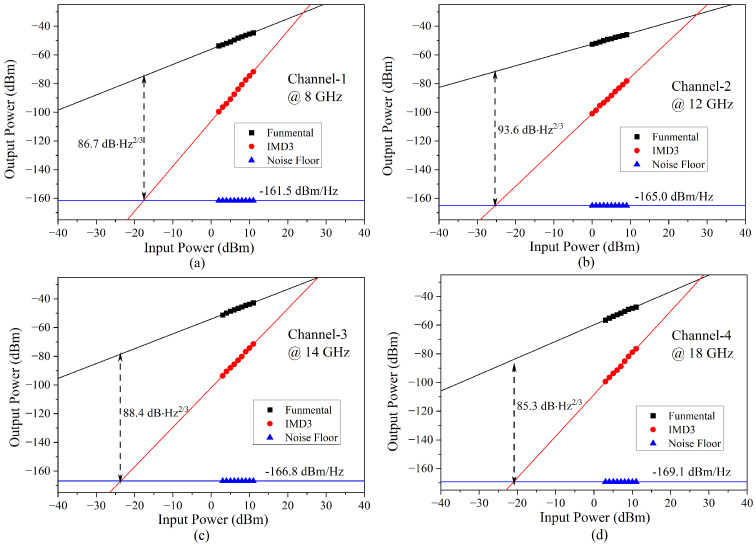
The SFDR characterization of four channels at (**a**) 8 GHz, (**b**) 12 GHz, (**c**) 14 GHz and (**d**) 18 GHz.

The SFDR indicating the linear range of the MWP receiving system was the measured. A two-tone signal with a frequency interval of 10 MHz was inputted with the change in the power. The recorded output power of the fundamental signal, the third-order intermodulation components and the noise floor for four channels of 8 GHz, 12 GHz, 14 GHz and 18 GHz are illustrated in Figure 10a–d, with SFDRs of 86.7 dB·Hz^2/3^, 93.6 dB·Hz^2/3^, 88.4 dB·Hz^2/3^ and 85.3 dB·Hz^2/3^, respectively. The SFDR performance was consistent with the SFDR levels of a common photonic integrated system [16]. The relatively high noise floor (−161.5 dBm/Hz) and small SFDR of Channel-1 at 8 GHz was mainly induced by the edge steepness of the OBPF. When the RF signal frequency was low, the unwanted sideband of the phase-modulated signal could not be completely filtered out. The residual sideband decreased the fundamental signal but increased the intermodulation component and noise, degrading the linearity of the MWP receiving system.

**Figure 11:**
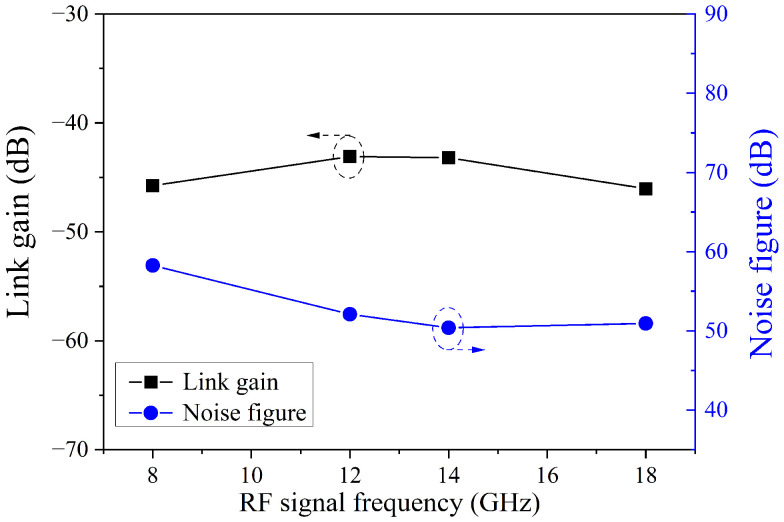
The gain and noise figure of the photonic chip-based MWP receiving system for four channels.

Based on the above measurement results, a gain of about −45 dB was obtained by calculating the difference between the output power and input power. The NF was about 50 dB, according to NF (dB) = 174 + N_out_ (dBm/Hz) − Gain(dB) [27]. The gain and noise figures of the four channels are shown in Figure 11. The small performance difference across the whole operation frequency band was attributed to the difference in the loss and noise floor at different frequencies, as shown in Figure 8 and Figure 10. The performance of the proposed multiband MWP system was comparable with that of other reported MWP systems [17]. It could be further improved by other measures [28] such as an increase in the output power of the laser source to reduce the coupling loss between the fiber and the waveguide by optimizing the coupling structure [29] and to enhance the power capacity of the PD [30].

## 4. Discussion

The preliminary experimental results demonstrated the feasibility of the proposed scheme for multiband RF signal receiving. The sideband filtering and the multiplexing of the multiband RF signals were implemented by a ring-assisted MZI-based OBPF and the multiband SSB + C signals were simultaneously detected by one PD. It demonstrated the compactness of the receiver. The scenario of the proposed scheme was designed so that the antennas were located at different sites as with, for example, a large naval ship, aircraft and distributed radar networks [31,32]. It could also be utilized for applications where the antenna is located near a central station or a post-signal processing system is connected to the antenna [14].

There is much work to be undertaken to enhance the function of the prototype scheme. First, a frequency down-conversion and post-processing were not included in the current stage. In our subsequent work, we intend to carry out research on multiband down-conversions by considering the LO signal modulation and spectrum processing in the optical domain. Second, the optically carried multiband signals were inputted to Port 1 and Port 2 via waveguide grating in the fabricated chip. The waveguide WDM could be directly connected to the OBPF, which would improve the multiband processing capacity [33]. Third, by increasing the number of micro-rings in the OBPF to increase the rectangular degree of the passband, the low frequency range of the receiver could be expanded [34]. Furthermore, the bandwidth of the current PD was relatively narrow, limiting the upper band of the receiving frequency signal. In addition, the multiple optically carried RF band signals were combined and detected by a single PD. To avoid being saturated, the PD should have a high-power processing capacity. By virtue of the rapid development of high-speed and high-power photodetectors on silicon photonics platforms, the high-frequency signals of the K-band, Ka-band and even broader bands can be received [30,31,32,33,34,35]. Last, but not least, the laser sources and phase modulators could be integrated with the OBPF and PD by advanced hybrid integration technology to realize the complete integration of MWP receiving systems [36,37].

## 5. Conclusions

In this paper, a silicon-based integrated MWP multiband signal receiving scheme was proposed and experimentally demonstrated. The multiband RF phase-modulated signals were processed by a ring-assisted MZI OBPF and then detected by a PD on the photonic integrated silicon chip. The OBPF had a periodical bandpass filtering response with a bandwidth of 60 GHz and an out-of-band suppression ratio of 16.6 dB. The PD had a 6 dB bandwidth of 25 GHz. The X- and Ku-bands, including four channels of 8 GHz, 12 GHz, 14 GHz and 18 GHz, were tested to verify the feasibility of the proposed scheme and the performance (including the receiving sensitivity, SFDR, gain and NF) were characterized. The measured results demonstrated the potential capacity of microwave photonic integration technology for multiband RF signal processing.

## Data Availability

Not applicable.
